# Transdermal Nicotine Poisoning: A Rare Case Report of Occupational Exposure

**DOI:** 10.3390/toxics11050464

**Published:** 2023-05-17

**Authors:** Jenny Becam, Edouard Martin, Gildas Pouradier, Natalia Doudka, Caroline Solas, Romain Guilhaumou, Nicolas Fabresse

**Affiliations:** 1 Laboratory of Pharmacokinetics and Toxicology, La Timone University Hospital, 264 Rue Saint Pierre, CEDEX 5, 13385 Marseille, France; 2 Intensive Care Unit, Laveran Military Teaching Hospital, 34 Boulevard Laveran, 13384 Marseille, France; 3 Department of Clinical Pharmacology and Pharmacovigilance, La Timone University Hospital, 264 Rue Saint Pierre, CEDEX 5, 13385 Marseille, France; 4 Institut de Neurosciences des Systèmes, Aix Marseille University, INSERM UMR 1106, 13385 Marseille, France; 5 Economic and Social Sciences of Health and Medical Information Processing, Aix Marseille University, INSERM UMR 1252, IRD, SESSTIM, 13385 Marseille, France

**Keywords:** toxicology, occupational medicine, nicotine, tobacco, cotinine, poisoning

## Abstract

We report a case of accidental nicotine intoxication following transdermal exposure in a 22-year-old man with no medical history, who worked in a company manufacturing e-liquids for electronic cigarettes. He accidentally spilled 300 mL of pure nicotine solution (>99%) on his right leg without wearing protective clothing or a mask. Less than a minute later, he experienced dizziness, nausea, and headaches, followed by painful burning sensations in the affected area. He immediately removed his pants and washed his leg thoroughly with water. He presented to the emergency department two hours later, where he exhibited a respiratory rate of 25 cpm, a heart rate of 70 bpm, headaches, abdominal pain, pallor, and vomiting. He recovered without specific treatment five hours post-intoxication. Plasma levels of nicotine, cotinine, and hydroxycotinine were measured five hours after exposure using liquid chromatography–mass spectrometry. The concentrations found were 447 ng/mL for nicotine, 1254 ng/mL for cotinine, and 197 ng/mL for hydroxycotinine. Nicotine is an alkaloid that can be highly toxic, with doses of 30–60 mg being potentially fatal. Transdermal intoxication is rare, with very few cases reported in the literature. This case highlights the risk of acute intoxication through cutaneous exposure to nicotine-containing liquid products and the need for protective clothing when handling such products in a professional context.

## 1. Introduction

Nicotine is an alkaloid found in the tobacco plant (*Nicotiana tabacum*). It is named after the documented medicinal use of tobacco leaves and seeds in 1560 by Jean Nicot de Villemain [1]. The compound was first isolated from tobacco in 1828 by Posselt and Reiman [2]. Nicotine amounts in plants range from 0.5 to 8% depending on the plant variety. Nicotine is considered to be a natural ingredient acting as a botanical insecticide in tobacco leaves. Nicotine is a potent stimulant. Its pharmacological mechanism of action is complex and involves interactions with multiple neurotransmitter systems in the brain. Nicotine binds to and activates nicotinic acetylcholine receptors (nAChRs) in the brain, which are widely distributed throughout the nervous system [3]. Activation of nAChRs leads to the release of several neurotransmitters, including dopamine, norepinephrine, and acetylcholine. Dopamine release in particular is thought to be responsible for the rewarding effects of nicotine and the development of addiction [4]. Nicotine increases dopamine levels in the mesolimbic pathway, which is involved in reward processing and motivation. This increase in dopamine release reinforces the behavior of smoking, leading to the development of dependence and addiction. Nicotine also affects other neurotransmitter systems in the brain, including the release of serotonin and endorphins. These effects contribute to the pleasurable sensations associated with smoking. While nicotine is commonly consumed through tobacco products, it is important to note that nicotine itself is a highly toxic substance. The lethal dose of nicotine can vary depending on factors such as age, weight, and health status, but in general, a dose of 30 to 60 mg can be fatal for adults [5]. Nicotine toxicity can occur through several routes of exposure, including ingestion, inhalation, and absorption through the skin. Ingesting or inhaling high doses of nicotine can cause acute poisoning, while chronic exposure to lower doses of nicotine can lead to long-term health problems. The symptoms of acute nicotine poisoning can appear rapidly and may include nausea and vomiting, abdominal pain and cramping, headache, dizziness and confusion, rapid heart rate and high blood pressure, weakness and tremors, seizures, respiratory failure, and coma [6]. In severe cases, acute nicotine poisoning can be fatal. Long-term exposure to nicotine can also cause a range of health problems, including increased risk of cardiovascular disease, respiratory problems, and cancer [7]. Nicotine can also have adverse effects on reproductive health, including reduced fertility and increased risk of birth defects.

While accidental ingestion of nicotine-containing products is a common cause of poisoning, occupational exposure to concentrated forms of nicotine can also pose a significant risk. In this case, report, we present the case of a 22-year-old man who suffered from nicotine poisoning after being exposed to a large amount of concentrated nicotine while working at an e-liquid factory. We describe the patient’s clinical presentation, management, and outcome, and discuss the potential implications for occupational health and safety. This case highlights the need for better awareness and preventive measures to reduce the risk of nicotine poisoning in the workplace.

## 2. Case Report

We present the case of a 22-year-old man, a smoker, with no prior medical history, who worked at an e-liquid factory. He accidentally spilled 300 mL of pure nicotine solution (>99%) on his right leg and was not wearing personal protective equipment (PPE) or a mask at the time. He immediately experienced dizziness, nausea, and headaches, and reported burning sensations and pain at the site of the spill on his leg. He removed his trousers and washed his leg with water. He presented to the emergency department two hours after the incident. On examination, the patient was alert and scored 15 on the Glasgow coma scale. His blood pressure was 111/56 mmHg, his heart rate was 70 bpm, his respiratory rate was 25 cpm, his oxygen saturation was 100% without oxygen supplementation, and his temperature was 36 °C. The patient complained of headaches and abdominal pain and vomited continuously. Neurological examination revealed chills and excessive salivation, while his pupils were intermediate and reactive. Cardiopulmonary examination did not show any abnormalities, while abdominal examination revealed diffuse tenderness and the presence of bowel sounds. There were no apparent cutaneous irritations on the right leg. His electrocardiogram (ECG) was normal, while arterial blood gas revealed a respiratory alkalosis with pH 7.59, pCO_2_ 18.1 mmHg, pO_2_ 126 mmHg, and lactate level of 6 mmol/L. His blood glucose level was 9.1 mmol/L. The patient received intravenous ondansetron for his nausea and paracetamol and morphine for pain management. He was also given 1.5 L of fluid resuscitation. His condition gradually improved over the next five hours without any specific treatment, and he was discharged from the hospital after 24 h. His lactate level was normalized to <2 mmol/L in 12 h. Five hours after the incident, the patient’s blood nicotine level was found to be 447 ng/mL, while his cotinine and hydroxycotinine levels were 1254 ng/mL and 197 ng/mL, respectively.

## 3. Materials and Methods

### 3.1. Reagents

Nicotine and 3-hydroxycotinine were provided by Cerillant (Sigma Aldrich, Munich, Germany). Cotinine and cotinine-D3 were purchased from LGC standard (Molsheim, France). Water purity was 18.2 mΩ/cm (Millipore, France). Pooled human plasma and whole blood were obtained from the Etablissement Français du Sang (EFS, Marseille, France). Buffer pH = 9 was prepared with Tritisol (Merck, Darmstadt, Germany). Acetonitrile, ammonia, and methanol were obtained from VWR International (Radnor, PA, USA).

### 3.2. Sample Preparation

Firstly, 250 µL of pH = 9 buffer was added to 250 µL of plasma, along with 25 µL of internal standard. Nicotine and its metabolites were then extracted via liquid–liquid extraction (LLE). Next, 1 mL of dichloromethane was added to the mixture, agitated for 20 min and centrifuged for 10 min at 14,500 rpm. The supernatant was then removed (aqueous phase) and the lower phase (organic) was transferred into a 5 mL glass tube and evaporated under a nitrogen flow for 10 min. The dry residue was reconstituted with 250 µL of mobile phase (90%A/10%B), 150 µL was transferred into a vial and 10 µL was injected into the chromatographic system.

Calibration standards (CS) were prepared from blank plasma spiked with the pure calibration solution in order to obtain a calibration range comprising 7 levels (10 ng/mL, 25 ng/mL, 50 ng/mL, 100 ng/mL, 250 ng/mL, 500 ng/mL and 1000 ng/mL) and 3 quality controls (QCs) (25 ng/mL, 100 ng/mL and 500 ng/mL).

### 3.3. Liquid Chromatography

Liquid chromatography was performed on an Acquity H-Class system (Waters, Milford, MA, USA). The compounds were eluted on a column BEH C18 (1.7 µm 2.1 mm × 50 mm; Waters, Milford, MA, USA), column temperature was set at 40 °C. Mobile phase A constituted water and acetonitrile (98/2; *v*/*v*) with ammonia 0.1%. Mobile phase B was pure acetonitrile. The gradient was as follows: 10%B for 1 min, linear gradient to 40%B for 3 min, back to 10%B in 0.1 min held for 1.4 min. Mobile phase flow rate was fixed at 0.4 mL/min. Total run time was 4.5 min.

### 3.4. Mass Spectrometry

The LC-MS/MS system used was a Quattro Premier triple quadrupole (Waters, Milford, MA, USA) with electrospray ionization (ESI). Capillary voltage was 3 kV, cone voltage 36 V, source temperature 120 °C, desolvation temperature 350 °C. Desolvation and cone gas flows were 850 L/h and 25 L/h, respectively. Collision gas was argon, gas flow was between 0.25 and 0.30 mL/min. Analyses were performed in multiple reaction monitoring (MRM) mode (Table 1).

### 3.5. Method Validation

This method was validated according to EMA guidelines for quantitation of nicotine, 3-hydroxycotinine and cotinine in plasma within the range 10–1000 ng/mL [8]. Details on the method validation including the limit of quantification, calibration range, and precision are provided in Supplementary Document S1.

## 4. Discussion

Nicotine poisoning is a well-known toxicological entity that can result from various routes of exposure, including ingestion, inhalation, and dermal absorption. Nicotine intoxication is frequent [9] but commonly related to accidental ingestion by children [10] or voluntary ingestion by adults [11]. Dermal absorption of nicotine can occur not only through occupational exposure but also through accidental or intentional use of nicotine-containing products, such as nicotine patches, gums, and sprays. Nicotine is known to easily cross the skin barrier; the bioavailability following transdermal exposure is around 68% [12]. The extent of skin contact and the pH of the solution are determining factors, since greater absorption occurs when nicotine is in the form of the free base [13]. This route of administration has been used in the treatment of tobacco addiction with transdermal patches. Despite the increasing use of electronic nicotine delivery systems (ENDS) along with nicotine containing e-liquids, nicotine poisoning via transdermal exposure remains quite rare.

Previous cases of transdermal nicotine exposure were reported among workers harvesting green tobacco as an occupational illness called “green-tobacco sickness” (10). Symptoms include headaches and dizziness in the afternoon following harvest and progress to abdominal pain, vomiting, and prostration in the evening. They last 12–24 h but recur in frequently exposed harvesters. Since then, several cases of acute poisoning following transdermal exposure to liquids containing nicotine have been documented [14,15,16,17]. Two well-documented cases of transdermal nicotine poisoning involved individuals who deliberately soaked their skin with a nicotine solution. In one case, a 45-year-old woman with scabies soaked her skin with a Black Leaf 40 solution (nicotine sulphate 40%) [17]. She experienced nausea and vomiting 15 min later, followed by lethargy, weakness, and abdominal cramps two hours later. Nicotine toxicokinetics revealed a sustained increase in plasma nicotine levels and a progressive increase in cotinine levels between 5 and 13 h after exposure. Plasma levels were between 200 and 400 ng/mL for nicotine and 400–800 ng/mL for cotinine. In the other case, a 34-year-old woman presented at the emergency department after a suicide attempt in which she spilled 120 mL of a 10% nicotine solution onto her body [16]. She experienced lethargy, tremors in all four extremities, vomiting, and atrial fibrillation with a heart rate of 90–100 bpm a few hours after exposure. Her nicotine serum level was 243 ng/mL on admission. In this case, report, we presented a case of occupational nicotine poisoning in a young man who spilled a large amount of concentrated nicotine on his skin while working at an e-liquid factory. The patient developed symptoms of dizziness, nausea, headache, and abdominal pain within minutes of exposure, and was found to have high levels of nicotine, cotinine, and hydroxycotinine in his blood. Our case is consistent with the current scientific literature on nicotine transdermal exposure resulting in poisoning. The patient experienced immediate symptoms (within 1 min) after the spill, which is a relatively rapid onset of symptoms compared to some other cases of nicotine poisoning. This may be due to the large amount of nicotine exposure and the fact that it was absorbed through the skin. The patient’s blood levels of nicotine and cotinine were very high compared to typical levels seen in smokers or other cases of nicotine poisoning (5–55 ng/mL) [18]. This may be due to the fact that the patient was exposed to a concentrated form of nicotine. A nicotine intoxication case study showed that the median nicotine blood concentration was significantly higher in non-survivors (up to 1600 ng/mL) versus survivors (under 800 ng/mL) [19]. In this case, the patient had a nicotine plasma concentration of 447 ng/mL, this was consistent with survival prognosis. Furthermore, the patient was a current smoker and thus presented a pharmacological tolerance to nicotine superior to a naïve subject. The patient’s nicotine blood level was measured 5 h after the intoxication. The blood level was probably higher within the first hour following poisoning, given that the nicotine half-life is short (40–120 min) [19]. The metabolic ratio between hydroxycotinine and cotinine has been used as a non-invasive indicator for CYP2A6 activity [20]. Following transdermal nicotine administration (nicotine patch users, *n* = 240), the ratio is about 0.5 ± 0.9 (mean ± SD) [21]. In this case, the metabolic ratio was much lower (0.2), highlighting a probable saturation of enzymatic metabolism by nicotine.

The management of nicotine poisoning resulting from transdermal exposure is similar to that of other routes of exposure, including supportive care, fluid resuscitation, and symptomatic relief. Atropine is used to treat cholinergic syndromes. In case of a seizure, benzodiazepines should be administered. Hemodialysis does not remove nicotine from the blood [22]. There are some specific considerations relating to dermal absorption, such as the need for immediate decontamination, removal of the nicotine source, and prevention of further exposure. In severe cases, ECMO (extracorporeal membrane oxygenation) may be considered, although these interventions are rarely necessary. This case highlights the potential dangers of working with concentrated nicotine and the importance of proper personal protective equipment (PPE) to minimize the risk of exposure, as recommended by the INRS (National Institute for Research and Safety).

## 5. Conclusions

Nicotine poisoning resulting from transdermal exposure is a rare but potentially serious condition that can occur in various settings, including occupational and non-occupational exposure to nicotine-containing products. Healthcare providers should be aware of the clinical features and management of nicotine poisoning and consider it in the differential diagnosis of patients with unexplained symptoms. Prevention of exposure to nicotine through appropriate handling and use of nicotine-containing products, as well as use of appropriate personal protective equipment, is key to avoiding this condition. Further research is needed to improve our understanding of the toxicology and epidemiology of nicotine poisoning resulting from transdermal exposure and develop better preventive and therapeutic strategies.

## Figures and Tables

**Table 1:** Cone voltage, *m/z* transitions and collision energy (CE).

Compounds	Cone Voltage (V)	Parent *m*/*z*	Quantification Fragment *m*/*z* (Collision Energy eV)	Confirmation Fragment *m*/*z* (Collision Energy eV)
Nicotine	32	163.12	117.06 (30)	130.07 (20)
Cotinine	36	177.22	80.01 (26)	98.05 (22)
3-hydroxycotinine	36	192.27	79.66 (18)	133.86 (36)
Cotinine-D3	36	180.21	79.94 (22)	101.01 (22)

## Data Availability

The data presented in this study are available on request from the corresponding author.

## Supplementary Materials

The following supporting information can be downloaded at: https://www.mdpi.com/article/10.3390/toxics11050464/s1, Document S1: Validation report.
