# Supplementary material for: Transdermal Nicotine Poisoning: A Rare Case Report of Occupational Exposure
Source: Toxics. 2023 May 17;11(5):464. doi: 10.3390/toxics11050464 (PMC10222450; doi:10.3390/toxics11050464)
Supplement: Supplementary file 1 [file toxics-11-00464-s001.zip › toxics-2370109-supplementary.pdf]

Validation report

1. Validation Method

1.1. Linearity

Six calibration curves were prepared with eight calibration standards (CS). Quantification was achieved by plotting the peak area ratios of uremic toxins (UT) to their internal standards (IS), and a weighting factor was determined. Back-calculated concentrations of the CS have to be within 85–115% of the nominal concentrations, except for the lower limit of quantification (LLOQ) a deviation of 20% was considered acceptable.

1.2. Lower Limit of Quantification (LLOQ)

The LLOQ was the lowest concentration achievable with an accuracy between 80 and 120 % and a coefficient of variation of ± 20 % obtained over six measurements at three different days. Three levels were evaluated: 25, 100 and 500 ng/mL.

1.3. Carry over

A blank sample was analyzed immediately after running three replicates of the highest CS to determine the carryover.

1.4. Accuracy and precision

The accuracy (bias, %) and precision (coefficient of variation, CV %) of the assay were determined for the three QC levels (25, 100 and 500 ng/mL) over three different days. Each day, 6 replicates of each QC were processed. The intra-assay bias and CV takes into account the variability of the 6 replicates within each validation day and the inter-assay bias and CV evaluates the variability between the three validation days. An accuracy within the range 85-115% of the nominal values and a precision with a CV of ±15% were required.

2. Validation results

2.1. Linearity

Table S1. Mean concentration of calibrant, bias, standard deviation and coefficient of variation.

		Theoretical concentrations (ng/mL)						
Molecules		**1000**	**500**	**250**	**100**	**50**	**25**	**10**
Nicotine	Mean (n=6)	1050	482	246	99	46	24	11
	Bias	-4,9%	3,7%	1,8%	1,3%	8,0%	5,9%	-5,4%
	SD	32,6	26,8	10,6	5,3	1,2	1,1	0,7
	CV	3,1%	5,6%	4,3%	5,4%	2,5%	4,7%	7,1%
Cotinine	Mean (n=6)	1033	483	232	101	48	24	10
	Bias	-3,3%	3,4%	7,0%	-1,3%	3,4%	4,8%	-2,8%
	SD	20,1	19,5	1,8	5,2	2,0	1,4	0,5
	CV	2,0%	4,0%	0,8%	5,2%	4,0%	5,7%	4,4%
3-hydroxycotinine	Mean (n=6)	1035	486	230	99	47	24	11
	Bias	-3,5%	2,8%	7,9%	0,9%	5,8%	5,2%	-8,0%
	SD	23,9	28,0	4,8	5,5	3,4	1,3	0,3
	CV	2,3%	5,8%	2,1%	5,5%	7,2%	5,5%	3,1%

2.2. Lower Limit of Quantification

Table S2. Lower limit of quantification (LLOQ).

	Intra-day (n=6)		
Molecules	LLOQ (ng/mL)	CV (%)	Bias (%)
Nicotine	10	2.0%	-7.8%
Cotinine	10	2.2%	-3.7%
3-hydroxycotinine	10	3.6%	2.5%

2.3. Carry over

No chromatographic peaks were evident for the analytes and for the internal standards when a blank was injected just after the highest point in the calibration.

2.4. Accuracy and precision

Table S3. Intra-day and inter-day accuracy and precision.

		Intra-day repeatability (n = 6)		Inter-day repeatability (n = 6)	
Molecules	Theoretical concentration (ng/mL)	CV (%)	Bias (%)	CV (%)	Bias (%)
Nicotine	25	14%	-7.7%	12%	-2.0%
	100	13%	-7.6%	9.9%	1.8%
	500	10%	1.1%	8.0%	2.4%
Cotinine	25	13%	7.8%	6.8%	0.6%
	100	4.0%	9.7%	9.5%	1.3%
	500	7.4%	5.9%	5.5%	2.4%
3-hydroxycotinine	25	11%	-2.8%	5.6%	-7.2%
	100	3.6%	-12%	7.2%	-1.0%
	500	8.1%	-0.3%	13%	0.9%
